# Different mechanisms of oxygenator failure and high plasma von Willebrand factor antigen influence success and survival of venovenous extracorporeal membrane oxygenation

**DOI:** 10.1371/journal.pone.0248645

**Published:** 2021-03-18

**Authors:** Tamara Steiger, Alois Philipp, Karl-Anton Hiller, Thomas Müller, Matthias Lubnow, Karla Lehle

**Affiliations:** 1 Department of Cardiothoracic Surgery, University Hospital Regensburg, Regensburg, Germany; 2 Department of Conservative Dentistry and Periodontology, University Hospital Regensburg, Regensburg, Germany; 3 Department of Internal Medicine II, University Hospital Regensburg, Regensburg, Germany; University of Bern, University Hospital Bern, SWITZERLAND

## Abstract

**Objective:**

Failure of membrane oxygenator (MO) function of venovenous extracorporeal membrane oxygenators (VV ECMO) remains problematic. The development of device-induced coagulation disorder (COD) or worsened gas transfer (WGT) necessitates a system exchange. The aim was to correlate von Willebrand factor antigen (vWF:Ag) with the predisposition to MO failure and mortality.

**Methods:**

Laboratory parameters (inflammation, coagulation) and ECMO-related data from 31 VV ECMO patients were analyzed before and after the first MO exchange. Study groups were identified according to the exchange reasons (COD, WGT) and the extent of vWF:Ag (low, ≤425%; high, >425%).

**Results:**

vWF:Ag remained unchanged after system exchange. High vWF:Ag was associated with systemic endothelial activation of older and obese patients with elevated SOFA score, increased norepinephrine and higher requirement of continuous renal replacement therapy without an effect on MO runtime and mortality. Including the mechanism of MO failure (COD, WGT), various patient group emerged. COD/low vWF:Ag summarized younger and less critically ill patients that benefit mainly from ECMO by a significant improvement of their inflammatory and coagulation status (CRP, D-dimers, fibrinogen) and highest survival rate (91%). Instead, WGT/high vWF:Ag presented older and more obese patients with a two-digit SOFA score, highest norepinephrine, and aggravated gas transfer. They benefited temporarily from system exchange but with worst survival (33%).

**Conclusions:**

vWF:Ag levels alone cannot predict early MO failure and outcome in VV ECMO patients. Probably, the mechanism of clotting disorder in combination with the vWF:Ag level seems to be essential for clot formation within the MO. In addition, vWF:Ag levels allows the identification different patient populations In particular, WGT/high vWF:Ag represented a critically ill population with higher ECMO-associated mortality.

## Introduction

All types of mechanical circulatory support devices, including venovenous extracorporeal membrane oxygenation (VV ECMO), bear a high risk of clot formation [1,2]. Both, thrombotic deposits on the surface of gas exchange fibers [3,4] as well as the extended clots inside the membrane oxygenators (MO) are discussed to impair the trans-membranous gas transfer and may eventually lead to MO failure [1,5,6]. An immediate exchange improved gas transfer performance and recovered critical coagulation parameters [1,4]. In general, two failure patterns were identified [1]: Worsened gas transfer (WGT) of the MO describes insufficient oxygenation and decarboxylation of patient blood. Device-induced coagulation disorder (COD) summarizes patients with critical changes of coagulation parameters (e.g. D-dimers, fibrinogen, platelets) and the development of acute clot formation within the pump head and MO. However, the underlying mechanisms remained unclear. Until now, there is no conclusive association between clot volume and time, anticoagulation regime or underlying disease of the patient [7].

The von Willebrand Factor (vWF) could be an important indicator protein for clot formation. ECMO support induced an acquired von Willebrand syndrome (AVWS) that is characterized by a loss of high molecular weight vWF (HMWvWF) due to high shear stress [8] and also a release of vWF of low activity from the endothelium in acute-phase response [9]. Furthermore, high accumulations of vWF in form of granular, cobweb-like and fiber-like structures were detected inside clotted MOs from ECMO patients with severe critical illness [7]. In these patients, it is obvious that shear stress activates vWF and accelerates platelet binding as an essential part in the process of clot formation [10–14].

In this study, we hypothesized that high plasma levels of the vWF antigen (vWF:Ag) of VV ECMO patients that required a system exchange due to WGT or COD are an indicator for impending clot formation inside the MO and therefore, might act as a predictor for MO failure and survival.

## Materials and methods

### Study design and study population

The Regensburg ECMO database was queried for consecutive patients with VV ECMO support (11/2017 to 11/2019, n = 128) and documented time dependent vWF:Ag courses. Only patients with at least one system exchange and a support duration of more than 2 days were included (n = 31). Parameters indicating the need for an exchange of the ECMO system were defined by Lubnow et al. [1]. Worsened gas transfer (WGT, 11/31, 35%) included patients that required a new system due to a decrease in CO_2_ transfer accompanied by an up-regulation of sweep gas flow and an increase in the partial pressure of carbon dioxide post MO (pCO_2_ post MO). The values improved with a new system (S1A Fig). Device-induced coagulation disorder (COD, 20/31, 65%) summarized all patients that presented any kind of detectable clotting disorder: Hyperfibrinolysis (n = 6; 19%) was defined by an increase in D-dimers and a decrease in fibrinogen (and platelet counts) (S1B Fig). Clot formation within the pump head (pump head thrombosis (PHT; n = 7; 23%) was identified by an increase in plasma free hemoglobin (fHb) and lactate dehydrogenase (LDH) (S1C Fig). A sudden increase in the pressure drop across the MO (dpMO) was an indicator for extended and occlusive clots within the MO (acute MO thrombosis, n = 7; 23%) (S1D Fig). All parameters improved after a system exchange.

Furthermore, vWF:Ag levels before an exchange were used to subdivide the exchange groups. Since almost all detected vWF:Ag levels were elevated a threshold value was defined that is the median of all values before exchange (425%). Two vWF:Ag groups were defined (vWF:Ag ≤425%, n = 16; vWF:Ag >425%, n = 15; hereinafter referred to as low and high vWF:Ag, respectively). Finally, four study groups were identified: low vWF:Ag/COD (n = 11), high vWF:Ag/COD (n = 9), low vWF:Ag/WGT (n = 5), high vWF:Ag/WGT (n = 6). Patient characteristics and ECMO data are summarized in Table 1.

**Table 1:** Study collective.

Parameter	all	COD	WGT	P-Value	Low vWF:Ag	High vWF:Ag	P-value	Low vWF:Ag / COD	High vWF:Ag / COD	Low vWF:Ag / WGT	High vWF:Ag / WGT	P-Value
Patients (n)	31	20	11	-	16	15	-	11	9	5	6	-
Age (years)	54 (41–67)	54 (34–63)	54 (43–68)	n.s.	44 (30–61)	60 (52–69)	**.008**	35 (21–59)^AB^	60 (54–70)^B^	54 (39–64)	60 (48–70)^A^	**sign**.
Males (n; %)	19; 61	10; 50	9; 82	n.s.	9; 56	10; 67	n.s.	5; 46	5; 56	4; 80	5; 83	n.s.
BMI (kg/m^2^)	27.8 (23.9–33.5)	26.4 (22.6–32.0)	27.8 (24.2–35.9)	n.s.	24.1 (21.3–27.8)	29.4 (27.8–35.9)	**.001**	23.4 (21.0–27.8)^CD^	28.9 (27.8–32.9)^BD^	26.0 (22.5–27.8)^AB^	35.3 (28.1–49.7)^AC^	**sign**.
SOFA-Score	10 (9–13)	10 (9–13)	12 (9–15)	n.s.	9 (8–10)	13 (10–15)	**.006**	9 (8–10)^A^	11 (9–15)	9 (8–13)	14 (12–15)^A^	**sign**.
LIS	3.3 (3.0–3.7)	3.3 (3.1–3.7)	3.3 (3.0–3.7)	n.s.	3.3 (3.0–3.7)	3.3 (3.0–3.7)	n.s.	3.3 (3.0–3.7)	3.3 (3.2–3.7)	3.3 (2.8–3.5)	3.3 (3.0–3.7)	n.s.
Classification of acute lung injury (n; %)*				n.s.			n.s.					n.s.
• Group 1: Pulmonary	26; 84	18; 90	8; 73		13; 81	13; 87		10; 91	8; 89	3; 60	5; 83	
• Group 2: Extra-pulmonary	3; 10	1; 5	2; 18		1; 6	2; 13		0; 0	1; 11	1; 20	1; 17	
• Group 3: Trauma	0; 0	0; 0			0; 0	0; 0		0; 0	0; 0	0; 0	0; 0	
• Group 4: Others	2; 7	1; 5	1; 9		2; 13	0; 0		1; 9	0; 0	1; 20	0; 0	
Anticoagulation (n; %)				n.s.			n.s.					n.s.
• Heparin	16; 52	9; 45	7; 64		8; 50	8; 53		5; 46	4; 44	3; 60	4; 67	
• Argatroban	15; 48	11; 55	4; 36		8; 50	7; 47		6; 55	5; 56	2; 40	2; 33	
MO model (n; %)				n.s.			n.s.					n.s.
• PLS	11; 36	6; 30	5; 46		5; 31	6; 40		2; 18	4; 44	3; 60	2; 33	
• ECC.05	7; 23	5;25	2; 18		5; 31	2; 13		4; 36	1; 11	1; 20	1; 17	
• Hilite7000LT	6; 19	5; 25	1; 9		3; 19	3; 20		3; 27	2; 22	0; 0	1; 17	
• Cardiohelp	6; 19	4; 20	2; 18		3; 19	3; 20		2; 18	2; 22	1; 20	1; 17	
• iLA-activve	1; 3	0; 0	1; 9		0; 0	1; 7		0; 0	0; 0	0; 0	1; 17	
Exchange-defining groups (n, %)				-			n.s.					-
• worsened gas transfer	11; 35	0; 0	11; 100		5; 31	6; 40		0; 0	0; 0	5; 100	6; 100	
• coagulation disorder	20; 65	20; 100	0; 0		11; 69	9; 60		11; 100	9; 100	0; 0	0; 0	
Subgroups coagulation disorder (n, %)				-			n.s.					-
• Hyperfibrinolysis	6; 19^§^	6; 30	0; 0		4; 36	2; 22		4; 36	2; 22	0; 0	0; 0	
• Pump head thrombosis	7; 23^§^	7; 35	0; 0		3; 27	4; 44		3; 27	4; 44	0; 0	0; 0	
• Trans-membrane pressure drop	7; 23^§^	7; 35	0; 0		4; 36	3; 33		4; 36	3; 33	0; 0	0; 0	
CRRT (n; %)	9; 29	5; 25	4; 36	n.s.	1; 6	8; 53	**.004**	0; 0^AB^	5; 56^B^	1; 20	3; 50^A^	**sign**.
HF ventilation (n; %)	7; 23	5; 25	2; 18	n.s.	4; 25	3; 20	n.s.	4; 36	1; 11	0; 0	2; 33	n.s.
Bleeding complications (n; %)	6; 19	4; 20	2; 18	n.s.	5; 31	1; 7	n.s.	4; 36^A^	0; 0^A^	1; 20	1; 17	n.s.

n.s., not significant. Definition of study groups: COD, coagulation disorder; WGT, worsened gas transfer; low vWF:Ag, vWF:Ag levels ≤425%; high vWF:Ag, vWF:Ag > 425%; combinations see in the text.

BMI, body mass index; SOFA, Sequential Organ Failure Assessment; LIS, Murray lung injury score; CRRT, continuous renal replacement therapy; HF ventilation, high frequency oscillatory ventilation.

* ECMO indications: 1, primary lung failure (bacterial, viral, fungal, aspiration pneumonia, H1N1 infection); 2, sepsis with secondary lung failure; 3, trauma with ARDS; 4, other pathologies (e.g. Pulmonary fibrosis, pulmonary hypertension, pulmonary emboli, extensive bronchiectasis, pulmonary bleeding, tracheal laceration).

^ABCD^ Pairwise comparisons interaction are considered significant with p-value ≤0.05.

^§^ Missing 35% are 11/31 MOs that were exchanged due to worsening of the gas transfer.

The primary endpoint was the effect of vWF:Ag levels on the clotting mechanism that predicts the need for an exchange. The secondary endpoint analyzed the effect of vWF:Ag levels on the MO run time and the outcome of the patients.

### ECMO management

The management of ECMO patients and the procedure to monitor MO performance has been previously described in detail [1]. Laboratory parameters were gathered on a daily routine (Institute for Clinical Chemistry and Laboratory Medicine, University Hospital Regensburg): e.g. activated partial thromboplastin time (aPTT), antithrombin III (ATIII), fibrinogen, D-dimers, C-reactive protein (CRP), lactate dehydrogenase (LDH), content of hemoglobin (Hb) as well as free hemoglobin (fHb), leukocyte and platelet count. Anticoagulation therapy with heparin or argatroban was administered according to requirements. In case there was no increased risk for bleeding, we aimed for an aPTT of 60sec. The median initial heparin dosage (ECMO day 1) was 6.2 [4.3–14] U/h/kg]. The median initial argatroban dosage was 1.4 [0.9–1.7] mg/h. MO performance characteristics (blood flow, sweep gas flow, CO_2_ transfer, O_2_ transfer, pressure drop across the MO, dpMO), blood gas analysis (BGA) results (*partial pressure of oxygen*, PO_2_; *partial pressure of carbon dioxide*, PCO_2_; each in arterial, venous and post MO blood samples), dosage of norepinephrine and anticoagulative therapy are gathered for the Regensburg ECMO database on a daily basis. O_2_ and CO_2_ transfer over the membrane are calculated based on formerly gathered BGA values [15]. Factor VIII, factor XIII, as well as vWF antigen (vWF:Ag test kit, Siemens Healthcare Diagnostics Products, Marburg, Germany) and vWF activity (Innovance vWF:Act, Siemens) were detected before and after a system exchange. vWF:Ag standard values range between 57.6 and 174.1% and vWF:Act standard values range between 47.8 and 173.2%. High molecular weight multimers were not analyzed.

### Data collection and statistical analysis

The study was approved by the Ethics Committee of the University of Regensburg (vote no. 20-1827-104). All data were collected in the Regensburg ECMO database from 11/2017 to 11/2019 (medical records from the University Hospital Regensburg) and fully anonymized for retrospective analysis (12/2019 to 6/2020). Need for informed consent was waived by the Ethics Committee as all devices are approved for clinical use, no personalized data and only routine laboratory parameters were used. Nominal and ordinal scaled data were depicted as frequencies and statistically analyzed using the χ^2^-test. Interval-scaled data were depicted as median and neighboring quartiles and statistically analyzed using Kruskal-Wallis, Wilcoxon, or Mann-Whitney-Test. Significance level was set to p = 0.05. For evaluation of different subgroups’ influence in general, the level of significance α was adjusted to α*(k) = 1-(1-α)^1/k^ with the Error Rates Method (k = number of paired tests performed). The software package IBM SPSS-Statistics 25.0 (IBM Corp., Armonk, NY, USA) was used for the statistical evaluation.

## Results

### Study population

vWF:Ag levels pre exchange were used to define the study groups: the median vWF:Ag level was 425% (vWF:Ag ≤425%, vWF:Ag >425%, hereinafter referred to as low and high vWF:Ag). As shown in Table 1, patients with high vWF:Ag levels were significantly older (p = 0.008), had a higher BMI (p = 0.001) and higher SOFA (Sequential Organ Failure Assessment) score (p = 0.006). Furthermore, they required continuous renal replacement therapy (CRRT; p = 0.004) more often compared to patients with low vWF:Ag levels. Cannula size and model were comparable, except for the choice of the drainage vessel (p = 0.038; S1 Table). Apart from that, all other patient characteristics and ECMO relevant parameters were comparable between both vWF:Ag groups.

11/31 (35%) and 20/31 (65%) of all MOs were exchanged due to WGT and COD respectively. According to the findings of Lubnow et al. [1], COD is a heterogeneous group consisting of hyperfibrinolysis, pump head thrombosis and trans-membrane pressure drop (S1 Fig). Of special interest were patients with high vWF:Ag and WGT that highlighted with old age, highest BMI, double-digit SOFA-scores and increased need for CRRT. Instead, low vWF:Ag and COD were dominant in young patients with normal BMI and less critical illness (SOFA).

### Technical complications demanding an exchange of the MO

MO complications leading to a system exchange can be assigned to two different failure patterns. WGT of the gas exchange membranes with insufficient oxygenation and decarboxylation of the patient’s blood and device-induced COD, which describes changes in coagulation parameters leading to clot formation in the MO and inducing procoagulant processes in the patient [1]. In order to analyze the varieties between WGT and COD, median laboratory and ECMO-related data over three days before the MO was exchanged were compared (Table 2).

**Table 2:** Ventilation-related data and laboratory parameters over 3 days before the MO was exchanged.

Parameter	Norm	COD	WGT	P-Value	Low vWF:Ag	High vWF:Ag	P-Value	Low vWF:Ag / COD	High vWF:Ag / COD	Low vWF:Ag / WGT	High vWF:Ag / WGT	P-Value
vWF:Ag [%]^S^*	58–174	413 (268–544)	423 (363–561)	n.s.	345 (257–377)	550 (506–578)	**< .001**	289 (242–369)^AB^	544 (505–581)^AC^	363 (294–395)^CD^	557 (473–587)^BD^	**sign**.
vWF:Act [%]*	48–173	213 (155–350)	256 (203–318)	n.s.	165 (115–216)	339 (264–397)	**< .001**	160 (99–209)^AB^	350 (275–399)^AC^	216 (151–279)^C^	274 (250–415)^B^	**sign**.
Hb [g/dL]	13.7–17.5	9.3 (8.9–10.1)	9.0 (8.6–9.8)	n.s.	9.3 (9.0–9.7)	9.0 (8.6–10.3)	n.s.	9.3 (9.1–9.4)	9.2 (8.5–10.4)	9.4 (8.5–9.9)	9.0 (8.4–10.3)	n.s.
Platelet count [/nL]	163–337	138 (93–190)	91 (66–176)	n.s.	133 (77–242)	124 (72–176)	n.s.	152 (108–192)	124 (85–199)	91 (46–315)	98 (57–162)	n.s.
aPTT [sec]	60	51 (43–61)	52 (45–58)	n.s.	47 (40–59)	54 (47–62)	n.s.	47 (39–58)	56 (47–67)	46 (42–63)	53 (47–55)	n.s.
ATIII [%]	80–112	86 (79–104)	92 (56–107)	n.s.	98 (79–110)	85 (57–96)	n.s.	101 (82–112)	83 (66–91)	95 (59–116)	91 (56–112)	n.s.
CRP [mg/L]	<3	149 (69–217)	181 (87–295)	n.s.	122 (49–184)	182 (113–306)	[.054]	113 (44–161)	182 (127–330)	181 (45–241)	202 (91–307)	n.s.
LDH [U/L]	<250	400 (334–575)	560 (431–637)	n.s.	394 (319–500)	573 (437–754)	**.006**	361 (294–500)^A^	573 (345–797)	431 (352–583)	602 (530–780)^A^	**sign**.
Leukocyte count [/nL]	4.0–10.0	10.0 (7.9–13.6)	10.8 (6.6–14.6)	n.s.	10.5 (7.5–14.2)	9.9 (7.0–11.7)	n.s.	10.1 (8.5–14.3)	9.7 (7.3–11.4)	11.1 (4.4–16.2)	10.6 (6.6–15.4)	n.s.
D-dimers [mg/L]^§^	<1	23 (12–34)	14 (8–34)	n.s.	20 (10–33)	26 (5–34)	n.s.	22 (11–35)	26 (9–34)	14 (9–30)	24 (4–35)	n.s.
Fibrinogen [mg/dL]	210–400	296 (156–480)	562 (456–621)	**.007**	254 (129–542)	482 (380–562)	**.045**	204 (123–321)^AB^	472 (320–488)^A^	571 (283–600)	530 (455–645)^B^	**sign**.
Factor VIII [%]	70–150	109 (80–185)	182 (86–290)	n.s.	99 (82–192)	146 (94–245)	n.s.	85 (70–164)	123 (106–220)	167 (85–253)	250 (81–307)	n.s.
Factor XIII [%]	60–146	34 (27–51)	38 (36–55)	n.s.	35 (27–39)	42 (30–55)	n.s.	30 (22–37)^AB^	55 (30–81)^B^	48 (36–61)^A^	38 (31–42)	n.s.
fHb [mg/L]	<50	42 (31–51)	40 (32–54)	n.s.	41 (30–51)	41 (32–51)	n.s.	44 (32–51)	35 (30–48)	39 (28–47)	45 (34–72)	n.s.
NE [mg/kg/h]	<0.01	0.01 (0.00–0.11)	0.09 (0.00–0.30)	n.s.	0.00 (0.00–0.05)	0.11 (0.00–0.40)	**.041**	0.0 (0.00–0.06)^A^	0.1 (0.00–0.25)	0.00 (0.00–0.07)^B^	0.30 (0.10–0.85)^AB^	n.s.
Blood flow [L/min]	-	3.0 (2.4–3.2)	3.2 (2.5–3.7)	n.s.	3.0 (2.3–3.2)	3.1 (2.8–3.5)	n.s.	3.0 (2.3–3.2)	3.0 (2.5–3.4)	2.5 (2.2–3.5)	3.2 (3.1–3.9)	n.s.
Gas flow [L/min]	-	5.5 (4.0–7.9)	9.0 (7.0–10.0)	**.040**	6.5 (4.1–8.0)	7.0 (4.0–10.0)	n.s.	6.0 (4.0–9.0)^A^	5.0 (3.5–6.5)^C^	7.0 (4.0–7.5)^B^	9.5 (9.0–10.5)^ABC^	**sign**.
CO_2_ transfer [mL/min]	-	172 (128–194)	208 (177–261)	**.029**	161 (142–204)	205 (158–243)	n.s.	152 (123–192)^A^	187 (130–214)^B^	177 (150–208)	243 (198–278)^AB^	n.s.
O_2_ transfer [mL/min]	-	155 (100–172)	150 (135–186)	n.s.	140 (91–173)	159 (140–186)	n.s.	162 (89–174)	151 (107–171)	135 (96–161)	173 (148–215)	n.s.
pCO_2_ post MO [mmHg]	35–45	38 (35–41)	39 (32–42)	n.s.	39 (35–42)	38 (33–40)	n.s.	39 (36–42)	37 (33–39)	39 (31–42)	39 (33–44)	n.s.
pO_2_ post MO [mmHg]	>250	375 (332–415)	353 (280–434)	n.s.	389 (323–444)	371 (325–392)	n.s.	375 (320–416)	374 (370–410)^A^	447 (336–493)	294 (212–370)^A^	n.s.

n.s., not significant.

sign., significant.

NE, norepinephrine.

^S^ Parameter defining the study groups.

* Quantification of parameter is limited at 600%.

^§^ Quantification of parameter is limited at 35mg/dL.

^ABC^ Pairwise comparisons interaction is considered significant with p-value ≤0.05.

WGT patients presented higher fibrinogen levels (p = 0.007) and required a higher gas flow (p = 0.040) with increased CO_2_ transfer (p = 0.029). A reduced platelet count was also striking, but without statistical significance. The content of hemoglobin, the fHb, aPTT, AT III, leukocyte count, D-dimers, the dose of norepinephrine as well as blood flow were equal in both groups.

### Impact of vWF:Ag plasma levels on laboratory and ECMO-related parameters

High vWF:Ag was accompanied by high vWF:Act (p<0.001) as well as altered inflammatory parameters: higher levels of LDH (p = 0.006) and fibrinogen (p = 0.045) and a tendency to higher CRP (p = 0.054). Additionally, patients with high vWF:Ag levels required a higher dose of norepinephrine (p = 0.041). Other parameters were not different between both vWF:Ag groups.

Including the vWF:Ag levels it was shown that patients in the COD group with low vWF:Ag levels were associated with lowest median values for vWF:Act, CRP, LDH, factor VIII, factor XIII, fibrinogen and CO_2_ transfer compared to the other subgroups (Table 2). However, only LDH and fibrinogen reached significance levels. These patients also needed less norepinephrine. For statistical comparison within the COD group, see Table 2. Another remarkable subgroup was the WGT group with high vWF:Ag levels. These patients presented highest levels of vWF:Act, LDH, CRP, gas flow, and CO_2_ transfer and highest dosage of norepinephrine, while the pO_2_ post MO was the lowest among all subgroups.

### vWF:Ag in the context of a MO exchange

A system exchange had no effect on vWF:Ag and vWF:Act independently of the exchange reason (COD or WGT) and the level of vWF:Ag (Table 3A and 3B). Statistical analysis compared the median values of laboratory and ECMO-relevant data (before, day of, post exchange) from the four subgroups. A system exchange led to significant changes in relevant parameters, particularly in COD patients with low vWF:Ag (Table 3A). Levels of LDH (p = 0.017) and fHb (p = 0.016) increased, while levels of fibrinogen (p = 0.033) and CO_2_ transfer (p = 0.026) were significantly lower on the exchange day. After exchange patients in this group presented lower CRP (p = 0.033), a shortened aPTT (p = 0.021), higher fibrinogen (p = 0.013), lower D-dimers (p = 0.005) and higher AT III (p = 0.021). Furthermore, fHb and pO_2_ post MO tended to improve after exchange (p = 0.062, each). pCO_2_ post MO tended to improve with the exchange (p = 0.053). In addition, the patients required less blood flow compared to the three days before exchange (p = 0.007). COD patients with high vWF:Ag showed less statistical benefit from a system exchange. Only CRP (p = 0.021), LDH (p = 0.038), D-dimers (p = 0.028) and fHb (p = 0.021) improved significantly after a system exchange. The leukocyte count increased minimally after exchange (p = 0.050) but remained approximately in the normal range. Gas exchange parameters were unremarkable.

**Table 3:** A. Ventilation-related data and laboratory parameters pre and post MO exchange in COD patients. B. Ventilation-related data and laboratory parameters pre and post MO exchange in WGT patients.

Parameter	Norm	Low vWF:Ag / COD			P-Value Pre/Post	P-Value Pre/ Exchange	P-Value Exchange/ Post	High vWF:Ag / COD			P-Value Pre/Post	P-Value Pre/ Exchange	P-Value Exchange/ Post
		Pre exchange	Exchange day	Post exchange				Pre exchange	Exchange day	Post exchange			
vWF:Ag [%]^S^*	58–174	289 (242–369)	354 (222–406)	281 (235–441)	n.s.	n.s.	n.s.	544 (505–581)	573 (507–599)	580 (470–599)	n.s.	n.s.	n.s.
vWF:Act [%]*	48–173	160 (99–209)	165 (132–213)	163 (122–237)	n.s.	n.s.	**.011**	350 (275–399)	328 (254–463)	331 (267–374)	n.s.	n.s.	n.s.
Hb [g/dL]	13.7–17.5	9.3 (9.1–9.4)	9.4 (8.6–10.1)	8.9 (8.4–9.7)	n.s.	n.s.	n.s.	9.2 (8.5–10.4)	8.5 (8.0–9.0)	9.6 (8.7–10.0)	n.s.	n.s.	n.s.
Platelet count [/nL]	163–337	152 (108–192)	152 (88–186)	132 (118–218)	n.s.	n.s.	n.s.	124 (85–199)	113 (85–178)	128 (88–217)	n.s.	n.s.	n.s.
aPTT [sec]	60	47 (39–58)	47 (38–48)	40 (37–44)	**.007**	n.s.	**.021**	56 (47–67)	56 (48–66)	54 (48–57)	n.s.	n.s.	n.s.
ATIII [%]	80–112	101 (82–112)	101 (93–110)	109 (101–114)	n.s.	n.s.	**.021**	83 (66–91)	91 (63–109)	99 (60–112)	n.s.	n.s.	n.s.
CRP [mg/L]	<3	113 (44–161)	60 (15–121)	34 (29–72)	**.045**	n.s.	**.033**	182 (127–330)	133 (100–312)	95 (62–229)	**.021**	**.021**	**.021**
LDH [U/L]	<250	361 (294–500)	486 (361–762)	412 (346–496)	n.s.	**.017**	n.s.	573 (345–797)	501 (370–1044)	461 (375–797)	n.s.	n.s.	**.038**
Leukocyte count [/nL]	4.0–10.0	10.1 (8.5–14.3)	11.6 (8.7–15.1)	10.2 (9.0–19.0)	n.s.	n.s.	n.s.	9.7 (7.3–11.4)	9.1 (6.2–13.5)	9.8 (6.6–15.4)	n.s.	n.s.	**.050**
D-dimers [mg/L]^§^	<1	22 (11–35)	28 (13–35)	8 (6–30)	**.008**	n.s.	**.005**	26 (9–34)	31 (11–35)	17 (8–20)	n.s.	n.s.	**.028**
Fibrinogen [mg/dL]	210–400	204 (123–321)	116 (63–237)	185 (143–256)	n.s.	**.033**	**.013**	472 (320–488)	330 (252–449)	353 (224–441)	n.s.	n.s.	n.s.
Factor VIII [%]	70–150	85 (70–164)	113 (55–145)	111 (62–152)	n.s.	n.s.	n.s.	123 (106–220)	118 (96–278)	162 (107–271)	n.s.	n.s.	n.s.
Factor XIII [%]	60–146	30 (22–37)	32 (20–34)	30 (24–33)	n.s.	n.s.	n.s.	55 (30–81)	48 (34–58)	58 (43–61)	n.s.	n.s.	n.s.
fHb [mg/L]	<50	44 (32–51)	56 (40–134)	47 (33–62)	n.s.	**.016**	[.062]	35 (30–48)	90 (35–802)	32 (25–85)	n.s.	n.s.	**.021**
NE [mg/kg/h]	<0.01	0.00 (0.00–0.06)	0.00 (0.00–0.01)	0.00 (0.00–0.00)	n.s.	n.s.	n.s.	0.10 (0.00–0.25)	0.06 (0.00–0.30)	0.00 (0.00–0.30)	n.s.	n.s.	n.s.
Blood flow [L/min]	-	3.0 (2.3–3.2)	2.6 (2.1–2.7)	2.5 (1.9–3.0)	**.007**	n.s.	n.s.	3.0 (2.5–3.4)	2.4 (2.2–2.9)	2.8 (2.0–3.5)	n.s.	n.s.	n.s.
Gas flow [L/min]	-	6.0 (4.0–9.0)	7.0 (3.0–9.0)	5.0 (3.0–6.0)	n.s.	n.s.	n.s.	5.0 (3.5–6.5)	4.0 (4.0–7.5)	5.0 (3.0–6.0)	n.s.	n.s.	n.s.
CO_2_ transfer [mL/min]	-	152 (123–192)	141 (117–160)	132 (101–186)	n.s.	**.026**	n.s.	187 (130–214)	151 (115–186)	156 (121–215)	n.s.	n.s.	n.s.
O_2_ transfer [mL/min]	-	162 (89–174)	140 (77–170)	124 (72–168)	n.s.	n.s.	n.s.	151 (107–171)	117 (98–181)	156 (109–171)	n.s.	n.s.	n.s.
pCO_2_ post MO [mmHg]	35–45	39 (36–42)	36 (33–40)	37 (33–37)	[.053]	n.s.	n.s.	37 (33–39)	36 (32–38)	36 (34–37)	n.s.	n.s.	n.s.
pO_2_ post MO [mmHg]	>250	375 (320–416)	383 (319–401)	454 (363–471)	**.050**	n.s.	[.062]	374 (370–410)	383 (324–451)	395 (304–446)	n.s.	n.s.	n.s.
Parameter	Norm	Low vWF:Ag / WGT			P-Value Pre/Post	P-Value Pre/ Exchange	P-Value Exchange/ Post	High vWF:Ag / WGT			P-Value Pre/Post	P-Value Pre/ Exchange	P-Value Exchange/ Post
		Pre exchange	Exchange day	Post exchange				Pre exchange	Exchange day	Post exchange			
vWF:Ag [%]^S^*	58–174	363 (294–395)	425 (305–459)	442 (424–562)	[.068]	n.s.	[.068]	557 (473–587)	592 (466–599)	580 (552–599)	[.068]	n.s.	n.s.
vWF:Act [%]*	48–173	216 (151–279)	261 (137–335)	270 (179–347)	n.s.	n.s.	n.s.	274 (250–415)	353 (217–465)	365 (226–532)	n.s.	n.s.	n.s.
Hb [g/dL]	13.7–17.5	9.4 (8.5–9.9)	9.6 (8.6–10.1)	9.5 (9.3–9.6)	n.s.	n.s.	n.s.	9.0 (8.4–10.3)	9.5 (8.1–10.7)	9.2 (8.5–10.1)	n.s.	n.s.	n.s.
Platelet count [/nL]	163–337	91 (46–315)	89 (40–247)	70 (34–244)	n.s.	n.s.	n.s.	98 (57–162)	114 (61–183)	118 (66–203)	**.028**	n.s.	n.s.
aPTT [sec]	60	46 (42–63)	49 (42–61)	53 (43–61)	n.s.	n.s.	n.s.	53 (43–61)	49 (45–52)	47 (44–50)	n.s.	n.s.	n.s.
ATIII [%]	80–112	95 (59–116)	99 (50–126)	98 (54–126)	n.s.	n.s.	n.s.	91 (56–112)	93 (68–107)	92 (69–117)	n.s.	n.s.	n.s.
CRP [mg/L]	<3	181 (45–241)	207 (22–242)	208 (15–295)	n.s.	n.s.	n.s.	202 (91–307)	194 (125–252)	173 (82–335)	n.s.	n.s.	n.s.
LDH [U/L]	<250	431 (352–583)	419 (363–633)	421 (294–598)	n.s.	n.s.	n.s.	602 (530–780)	573 (515–735)	577 (472–701)	n.s.	n.s.	n.s.
Leukocyte count [/nL]	4.0–10.0	11.1 (4.4–16.2)	12.7 (6.8–18.6)	12.7 (7.6–19.2)	n.s.	n.s.	n.s.	10.6 (6.6–15.4)	12.2 (9.1–16.2)	9.9 (7.8–15.9)	n.s.	n.s.	n.s.
D-dimers [mg/L]^§^	<1	14 (9–30)	17 (10–28)	7 (5–13)	**.043**	n.s.	**.043**	24 (4–35)	35 (5–35)	21 (7–35)	n.s.	n.s.	n.s.
Fibrinogen [mg/dL]	210–400	571 (283–600)	433 (249–561)	425 (235–653)	n.s.	[.080]	n.s.	530 (455–645)	456 (355–706)	425 (342–726)	n.s.	n.s.	n.s.
Factor VIII [%]	70–150	167 (85–253)	226 (120–341)	231 (94–344)	n.s.	n.s.	n.s.	250 (81–307)	231 (79–354)	272 (132–390)	n.s.	n.s.	n.s.
Factor XIII [%]	60–146	48 (36–61)	54 (35–75)	70 (39–87)	n.s.	n.s.	n.s.	38 (31–42)	33 (20–42)	35 (23–49)	n.s.	n.s.	n.s.
fHb [mg/L]	<50	39 (28–47)	35 (20–71)	34 (26–40)	n.s.	n.s.	n.s.	45 (34–72)	42 (34–67)	36 (32–71)	n.s.	n.s.	n.s.
NE [mg/kg/h]	<0.01	0.00 (0.00–0.07)	0.02 (0.00–0.25)	0.02 (0.00–0.06)	n.s.	n.s.	n.s.	0.30 (0.10–0.85)	0.50 (0.16–1.08)	0.08 (0.00–0.50)	n.s.	n.s.	n.s.
Blood flow [L/min]	-	2.5 (2.2–3.5)	2.9 (2.4–3.8)	3.1(2.4–3.9)	n.s.	n.s.	n.s.	3.2 (3.1–3.9)	3.5 (2.6–4.3)	2.9 (2.3–4.1)	n.s.	n.s.	n.s.
Gas flow [L/min]	-	7.0 (4.0–8.0)	10.0 (5.0–11.0)	6.0 (4.5–7.0)	n.s.	n.s.	n.s.	9.5 (9.0–10.5)	11.0 (10.8–11.3)	6.5 (3.5–11.3)	n.s.	n.s.	[.066]
CO_2_ transfer [mL/min]	-	177 (150–208)	193 (107–238)	198 (162–208)	n.s.	n.s.	n.s.	243 (198–278)	154 (86–209)	229 (164–318)	n.s.	**.046**	**.046**
O_2_ transfer [mL/min]	-	135 (96–161)	141 (134–156)	125 (110–165)	n.s.	n.s.	n.s.	173 (148–215)	145 (101–199)	169 (114–241)	n.s.	n.s.	n.s.
pCO_2_ post MO [mmHg]	35–45	39 (31–42)	41 (38–47)	36 (31–39)	n.s.	**.042**	[.068]	39 (33–44)	43 (39–50)	36 (30–43)	n.s.	[.074]	**.028**
pO_2_ post MO [mmHg]	>250	447 (336–493)	364 (187–466)	354 (332–468)	n.s.	n.s.	n.s.	294 (212–370)	213 (121–311)	289 (271–349)	n.s.	n.s.	n.s.

n.s., not significant.

NE, norepinephrine.

^S^ Parameter defining the study groups.

* Quantification of parameter is limited at 600%.

^§^ Quantification of parameter is limited at 35mg/dL.

A system exchange due to WGT and low vWF:Ag only presented a significant reduction of D-dimers (p = 0.043) while pCO_2_ post MO tended to be lower after MO exchange. The latter increased significantly before MO exchange (p = 0.042). An increase in gas flow before and a decrease after exchange was shown, but without statistical significance. CO_2_ and O_2_ transfer were inconspicuous. In contrast, patients with WGT and high vWF:Ag benefited significantly from a system exchange. Initially low platelet counts normalized or even increased after the MO replacement (p = 0.028). Other WGT specific gas data improved after exchange—patients required less gas flow (p = 0.066). In addition, worsened CO_2_ transfer and increased pCO_2_ post MO improved significantly after exchange (p = 0.046 and p = 0.028, respectively).

As shown in S2 Table, there was no difference in the need for transfusion of red blood cells, fresh frozen plasma and platelet concentrates.

### MO runtime and vWF:Ag plasma levels

High levels of vWF:Ag tended to shorten MO runtime (p = 0.054; Table 4). This effect was most pronounced in the COD group (pairwise comparison see Table 4). However, the differences (comparing all subgroups) were not significant due to the high scatter with small number of cases. The level of vWF:Ag had no effect on the total duration of ECMO independently of the exchange reasons (Table 4).

**Table 4:** MO runtime and outcome.

Parameter	all	COD	WGT	P-Value	Low vWF:Ag	High vWF:Ag	P-Value	Low vWF:Ag / COD	High vWF:Ag / COD	Low vWF:Ag / WGT	High vWF:Ag / WGT	P-Value
Patients (n)	31	20	11	-	16	15	-	11	9	5	6	-
First MO’s runtime (days)	9 (7–14)	9 (6–15)	9 (6–18)	n.s.	11 (8–19)	7 (5–11)	[.054]	11 (9–19)^A^	7 (5–10)^A^	9 (7–21)	10 (6–14)	n.s.
Duration of ECMO-support (days)	22 (16–36)	24 (16–42)	19 (15–28)	n.s.	27 (19–42)	17 (15–36)	n.s.	26 (19–44)^A^	17 (14–43)	27 (18–64)	17 (14–32)^A^	n.s.
Successful weaning of ECMO (n; %)	21; 68	17; 85	4; 36	**.006**	12; 75	9; 60	n.s.	10; 91^AB^	7; 78	2; 40^A^	2; 33^B^	**sign**.
Discharge from hospital (n; %)	20; 65	16; 80	4; 36	**.015**	12; 75	8; 53	n.s.	10; 91^AB^	6; 67	2; 40^A^	2; 33^B^	n.s.

n.s., not significant.

sign., significant.

^AB^ Pairwise comparisons interaction is considered significant with p-value ≤0.05.

### Outcome in case of worsened gas transfer or coagulation disorder

As shown in Table 4, patients whose first MO was changed due to COD were more often successfully weaned (p = 0.006) and significantly more patients discharged hospital (p = 0.015). In contrast, the level of vWF:Ag was not an indicator for successful weaning or discharge from hospital. Subgroup analysis disclosed highest successful weaning rates (91%, significant) and survival rates (91%, not significant) for patients with COD and low vWF:Ag. In the WGT group there was a 60% (3/5) mortality with low vWF:Ag and a 67% (4/6) mortality with high vWF:Ag.

## Discussion

While the implantation of an ECMO system resulted in a decrease of vWF:Ag and vWF:Act [9,16], a system exchange had no effect on the levels of vWF:Ag or vWF:Act. Furthermore, the exchange reason (COD, WGT) had no effect on vWF:Ag or vWF:Act. However, the level of vWF:Ag combined with the exchange reason identified different patient populations. Patient populations were defined regarding state of critical illness, inflammation and coagulation status, benefit of a system exchange, MO runtime, and mortality. Low vWF:Ag levels and COD represent younger patients with minor acute-phase response, on trend prolonged MO runtime and highest survival rates. In contrast, high vWF:Ag levels and WGT define critically ill patients with the highest mortality rate and with severe respiratory failure requiring an escalation of trans-membranous gas transfer.

Elevated vWF:Ag levels in ECMO patients, a decrease within one day on ECMO and normalization after explant of ECMO have been reported by Tauber et al. [16]. vWF:Ag is elevated during acute-phase response [17] due to endothelial activation and injury in acute and chronic inflammation [18]. Almost all of the vWF:Ag levels before a system exchange were elevated. Therefore, we subdivided our patient population due to the median vWF:Ag level of 425% to get comparable group sizes. A similar approach has been described in other studies [19,20]. A threshold value of ≥450% was associated with a greater risk of developing acute lung injury (ALI) or acute respiratory distress syndrome (ARDS) in non-septic and septic patients [19,20]. It has been associated with adverse outcomes (mortality, duration of unassisted ventilation, organ failure) in patients with established ALI/ARDS [19]. Therefore, it was not surprising that all patients requiring VV ECMO presented elevated vWF:Ag and vWF:Act levels [9,16]. An adjustment to the threshold value of 450% did not change our results. We concluded that high vWF:Ag levels in VV ECMO patients indicate a severe inflammatory response and reflect the degree of critical illness of the patient.

In our study, patients with vWF:Ag >425% were of older age with elevated BMI and in a state of critical illness (increased SOFA score, need for CRRT). The elevated vWF-Ag reflected an increased acute phase response accompanied by increased levels of CRP and fibrinogen. Correlations between CRP and vWF:Ag in ECMO patients have also been described by Tauber et al. [16] but without differentiation of the vWF:Ag level. Furthermore, high vWF:Ag was associated with increased LDH levels indicating distinct organ damage. Under these conditions, the runtime of the first MO tended to be shorter without affecting the patients’ outcome. We assume that the severe critical illness with high vWF:Ag levels promote protein as well as platelet and leukocyte adhesion to the foreign surface of the MO [10–14] and progress clot formation inside the MO that manifested in a reduced MO runtime and early MO failure.

The differentiation of reasons for a system exchange (WGT, COD) resulted in differences regarding inflammatory and coagulation markers, gas transfer capability, run time of the MO and mortality. Patient characteristics were comparable. Exchange criteria were identified previously [1]. Within 3 days before an exchange of the MO, the WGT group presented less platelets and required higher gas flow to meet the increased demand of CO_2_ elimination. The COD group is heterogeneous including all subgroups with detectable clot formation within the system: Sudden increases in fHb levels identified clots within the pump head (PHT). An increase in the dpMO indicated the growth of clots within the MO resulting in the occlusion of the MO (acute oxygenator thrombosis). The typical alterations in specific coagulation parameters (e.g. d-dimers, fibrinogen, platelet counts) described an impending clotting disorder (hyperfibrinolysis) [1]. Analysis of the subgroups failed due to less group size. The COD group presented a significant loss in fibrinogen levels, while the gas exchange capability of the MO remained unchanged. Despite these differences, the MO runtime as well as total ECMO supporting time was comparable between COD and WGT. However, WGT (vs. COD) resulted in less frequency of successful weaning (36% vs. 85%, p = 0.006) and reduced survival (36% vs. 80%, p = 0.015). We assume that the failing mechanism that ended in a system exchange was different comparing WGT and COD. Thrombotic deposits in membrane lungs are probably responsible for the deterioration in the gas exchange performance of artificial lungs [6]. Different mechanisms are possible. Already the adhesion of proteins and binding of blood cells onto the gas exchange fibers may impede its performance (represents the WGT group in our study). Furthermore, adhesive proteins initiate extensive clotting inside the MO and reduce the perfused area (represented by COD in our study) [6]. In both cases, the involvement of the vWF could be conceivable—a linker for blood cells (adhesion/ interconnection of platelets and leukocytes) or as a shear-dependent component in clot formation [7,10–12]. In future studies, the relationship between the different clot mechanisms (COD, WGT) and the actual clot volume and the clot composition within the MOs is to be investigated [3,4,7].

Including the inflammatory component—verified by high and low vWF:Ag—patients from the COD/ low vWF:Ag group presented moderate critical illness (younger, lower SOFA score) without systemic inflammation (low CRP, LDH, fibrinogen). The mechanism of a device-induced COD seems to be reversible by exchanging the system. Our data show significant reduction of CRP, aPTT and D-dimers and an increase of AT III in these patients. After the system exchange, flow inside the MO normalized. Therefore, triggers of coagulation and inflammation are controllable temporarily. Surprisingly, 91% of these patients could be weaned successfully from ECMO and all of these patients discharged hospital. In contrast, in COD patients with high vWF:Ag levels a system exchange showed limited efficiency regarding inflammatory and coagulation parameters. Furthermore, the insertion of a new MO did not influence oxygenation and decarboxylation parameters. However, initially high levels of CRP and D-dimers decreased significantly after the system exchange, indicating that the local inflammation and hyperfibrinolysis could be remedied. So, clotting inside the MO in COD patients with high vWF:Ag is not only device-induced, but also patient-induced due to a pre-existing systemic inflammation. According to our data we suggest, that one has to differentiate between moderate inflammation (vWF:Ag ≤425%) due to foreign surface activation inside the MO and severe inflammation (vWF:Ag >425%) which originates mostly in a pre-existing status of systemic inflammation. Severe inflammation is only to a small amount induced by the MO itself. High vWF:Ag levels enhance the local inflammatory response to the foreign surface of the MO leading to clot formation and early MO failure. The procoagulant tendency of vWF depends on the predominant shear rate [21,22]. Therefore, the reduction in MO runtime in COD patients with high vWF:Ag levels might be due to alterations of blood flow. Moreover, increased local shear rates which favor the vWF-mediated clot formation and also local inflammation [23] might be considered as possible reasons. Furthermore, high levels of fHb on the exchange day normalized within one day. The dominance of this parameter is based on the fact that 35% of the COD patients presented a proven PHT. By replacing the clotted pump head, fHb decreased immediately [1].

In the WGT group with low vWF:Ag, the raise of gas flow before and the subsequent decrease after exchange as well as the alterations of pCO_2_ post MO mainly reflected worsening of gas transfer capability [1]. The additional reduction of D-dimers indicated the presence of thrombotic deposits within the used MO that disappeared after implantation of a new MO [24,25]. In contrast, a system exchange did not affect the other relevant parameters in the patient population. In particular, high levels of the inflammatory CRP remained high. The latter observation could also indicate that WGT has a different mechanism of clot formation within the system compared to COD (see above). In particular, the subgroup with WGT and high vWF:Ag identified critically ill patients with highest SOFA scores, obese BMI, elevated requirement of CRRT, high dosage of norepinephrine, and increased need for extracorporeal oxygenation. In contrast, a system exchange did not improve inflammation or coagulation within the WGT group with low vWF:Ag. However, the gas exchange situation improved and the system-induced thrombocytopenia regenerated significantly. The latter was also described by Lubnow et al. [1]. Despite increased pre-existing systemic inflammation (high CRP) MO runtime was not impaired with regard to vWF:Ag levels. We speculated that these patients, who tend to be in a severe septic status with thrombotic microangiopathy lack the ability to clot inside the MO as a result of disseminated intravascular coagulation (DIC) [26,27]. Furthermore, since extended protein adhesion to the gas exchange membrane reduces gas exchange capacity, there are still bedside coping strategies such as increasing gas flow or increasing blood flow to preserve sufficient gas transfer [28,29]. Furthermore, the gas transfer recovered or improved after a system exchange—this is a clear indication that protein or cellular deposits on the gas exchange membrane are responsible for the worsening of the gas transfer. Despite the improvement of the gas transfer rates with a new system, 33% of these patients died on ECMO.

Limitations of this study include its retrospective and single-center character and inclusion of only 31 VV ECMO patients with even smaller subgroups. Furthermore, the data evaluation was incomplete for vWF:Ag, vWF:Act, factor VIII and factor XIII that prevented a clear correlation with other parameters. The threshold of vWF:Ag (425%) used exceeds the standard value maximum of 174%. Therefore both study groups though indicated with “high” or “low” vWF:Ag levels represent patients with vWF:Ag levels high above standard values.

## Conclusion

While typical coagulation parameters improve significantly when a system exchange is initiated, the vWF:Ag plasma levels of VV ECMO patients are not an indicator for impeding clot formation. Probably, the mechanism of clotting disorder in combination with the vWF:Ag level seems to be essential for the development of clots within the ECMO system. Nevertheless, the vWF:Ag level may provide information about the reversibility of local inflammatory processes inside the MO. Overall, the quantification of vWF:Ag levels alone neither can predict patient’s outcome nor can it predict early MO failure in VV ECMO patients.

## Supporting information

S1 Fig Parameters indicating need for system exchange. (PDF)

S1 Table Cannulation specifications of the study cohort. (PDF)

S2 Table Transfusion of red blood cell concentrates, fresh frozen plasma and platelet concentrates. (PDF)

S1 Data (XLSX)
