# Supplementary material for: Different mechanisms of oxygenator failure and high plasma von Willebrand factor antigen influence success and survival of venovenous extracorporeal membrane oxygenation
Source: PLoS One. 2021 Mar 18;16(3):e0248645. doi: 10.1371/journal.pone.0248645 (PMC7971568; doi:10.1371/journal.pone.0248645)
Supplement: S1 Fig — (PDF) [file pone.0248645.s001.pdf]

S1 Fig. Parameters indicating need for system exchange

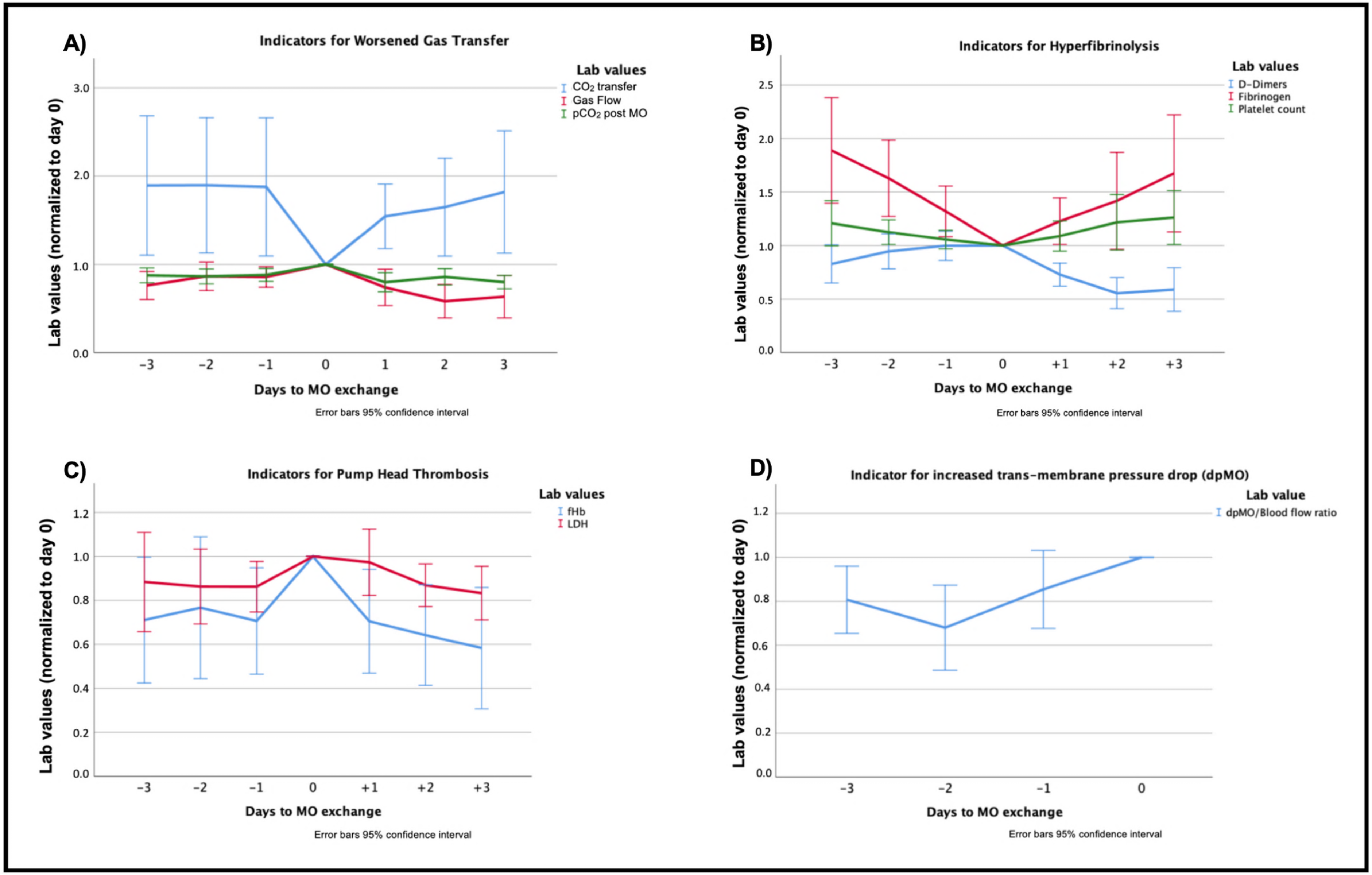

Data are normalized to day 0 (day of system exchange).
A) Indicators for worsened gas transfer:
35% of the included MOs (11/31) had been changed for reason of WGT. WGT manifested through increase of partial pressure of carbon dioxide after the MO (pCO_2 post MO; day -2: 0.83[0.69-1.10]; day 0: 1.00; p=0.036), decrease after exchange (day 0: 1.00; day +1: 0.77[0.50-1.00]; p=0.003), loss of CO_2 transfer (day -1: 1.42[0.90-4.85]; day 0: 1.00; p=0.024), followed by post-exchange reuptake (day 0: 1.00; day +3: 1.39[0.99-3.59]; p=0.026). Gas flow increase over the last 72h before exchange (day -3: 0.73[0.42-1.17]; day 0: 1.00; p=0.304), reduction over 72h after exchange (day 0: 1.00; day +3: 0.50[0.27-1.09]; p=0.047). Loss of O_2 transfer (day -1: 1.05[0.49-1.78]; day 0: 1.00; p=1.000) and decrease of partial pressure of oxygen after the MO (pO2 post MO; day -1: 1.17 [0.83-3.06]; day 0: 1.00; p=0.489) are not significant.

B) Indicators for hyperfibrinolysis:
30% (6/20) of the MOs in the COD-group were exchanged due to HF. Significant loss of fibrinogen before exchange (day -3: 2.15[1.39-4.43]; day 0: 1.00; p=0.034). Increase of platelet count after exchange (day 0: 1.00; day +3: 1.81[1.33-2.64]; p=0.007). Increasing D-dimers before exchange (day -3: 0.50[0.26-0.89]; day 0; 1.00; p=0.105).

C) Indicators for pump head thrombosis:
7 MOs (35%) had been exchanged due to acute formation of PHT. PHT was diagnosed when fHb increased to a multiple of its base value (day -3: 0.05[0.02-0.53]; day 0: 1.00; p=0.002). After exchange, there is no more analytical proof for hemolysis (day 0: 1.00; day +3: 0.08[0.01-0.60]; p=0.011). Besides, LDH levels increased over 72h before PHT (day-3: 0.52[0.35-0.71]; day 0: 1.00; p=0.001) and decreased over the next 72h after exchange (day 0: 1.0; day +3: 0.66[0.40-0.87]; p=0.017).

D) Indicators for trans-membrane pressure drop:
35% (7/20) of the MOs were exchanged because pressure drop in the MO increased, accompanied by reduction of blood flow. dpMO levels after system exchange are not displayed. There is an increase in trans-membrane pressure drop starting 72h before exchange (day -3: 0.60[0.35-0.80]; day 0: 1.00; p=0.037).
