# Supplementary material for: Different mechanisms of oxygenator failure and high plasma von Willebrand factor antigen influence success and survival of venovenous extracorporeal membrane oxygenation
Source: PLoS One. 2021 Mar 18;16(3):e0248645. doi: 10.1371/journal.pone.0248645 (PMC7971568; doi:10.1371/journal.pone.0248645)
Supplement: S1 Table — (PDF) [file pone.0248645.s002.pdf]

S1 Table. Cannulation specifications of the study cohort.

Parameter	all	COD	WGT	P-Value	Low vWF:Ag (≤425%)	High vWF:Ag (>425%)	P-value	Low vWF:Ag / COD	High vWF:Ag / COD	Low vWF:Ag / WGT	High vWF:Ag / WGT	P-Value
Patients (n)	31	20	11	-	16	15	-	11	9	5	6	-
Outflow cannula (n; %)				n.s.			n.s.					n.s.
- Maquet[§] 23/ 38	13; 42	8; 40	5; 45		5; 31	8; 53		3; 27	5; 55	2; 40	3; 50	
- Maquet[§] 21/ 38	13; 42	10; 50	3; 27		6; 38	7; 47		6; 55	4; 44	0; 0	3; 50	
- ParaGlide[$] 23/ 33	3; 10	1; 5	2; 18		3; 19	0; 0		1; 9	0; 0	2; 40	0; 0	
- Avalon[∔] 20/ 31	1; 3	1; 5	0; 0		1; 6	0; 0		1; 9	0; 0	0; 0	0; 0	
- NovaPort Twin[△] 24/ 27	1; 3	0; 0	1; 9		1; 6	0; 0		0; 0	0; 0	1; 0	0; 0	
Drainage vessel (n; %)				n.s.			**.038**					n.s.
- Femoral vein ($V_{fl/fr}$)	27; 87	18; 90	9; 82		12; 75	15; 100		9; 82	9; 100	3; 60	6; 100	
- Jugular vein (V_{jr})	4; 13	2; 10	2; 18		4; 25	0; 0		2; 18	0; 0	2; 40	0; 0	
Inflow cannula (n; %)				n.s.			n.s.					n.s.
- Maquet[§] 21/ 23	2; 6	1; 5	1; 9		1; 6	1; 7		1; 9	0; 0	0; 0	1; 17	
- Maquet[§] 19/ 15	15; 48	11; 55	4; 36		6; 38	9; 60		4; 36	7; 77	2; 40	2; 33	
- Maquet[§] 17/ 15	7; 23	5; 25	2; 18		3; 19	4; 27		3; 27	2; 22	0; 0	2; 33	
- Maquet[§] 17/ 23	1; 3	0; 0	1; 9		0; 0	1; 7		0; 0	0; 0	0; 0	1; 17	
- Maquet[§] 15/ 15	1; 3	1; 5	0; 0		1; 6	0; 0		1; 9	0; 0	0; 0	0; 0	
- ParaGlide[$] 23/ 33	3; 10	1; 5	2; 18		3; 19	0; 0		1; 9	0; 0	2; 40	0; 0	
- Avalon[∔] 20/ 31	1; 3	1; 5	0; 0		1; 16	0; 0		1; 9	0; 0	0; 0	0; 0	
- NovaPort Twin[△] 24/ 27	1; 3	0; 0	1; 9		1; 16	0; 0		0; 0	0; 0	1; 20	0; 0	
Return vessel (n; %)				n.s.			n.s.					n.s.
- Femoral vein ($V_{fl/fr}$)	1; 3	0; 0	1; 9		1; 6	0; 0		0; 0	0; 0	1; 20	0; 0	
- Jugular vein (V_{jr})	30; 97	20; 100	10; 91		15; 94	15; 100		11; 100	9; 100	4; 80	6; 100	

n.s., not significant

[§]Maquet, Hirrlingen, Germany

[$]ParaGlide double lumen cannula, Chalice Medical Ltd., Nottinghamshire, UK

[∔]Avalon Elite Bi-Caval Dual Lumen Catheter, Maquet, Hirrlingen, Germany

[△]NovaPort Twin Dual Lumen Catheter, Novalung, Heilbronn, Germany
