# Supplementary material for: Different mechanisms of oxygenator failure and high plasma von Willebrand factor antigen influence success and survival of venovenous extracorporeal membrane oxygenation
Source: PLoS One. 2021 Mar 18;16(3):e0248645. doi: 10.1371/journal.pone.0248645 (PMC7971568; doi:10.1371/journal.pone.0248645)
Supplement: S2 Table — (PDF) [file pone.0248645.s003.pdf]

S2 Table. Transfusion of red blood cell concentrates, fresh frozen plasma and platelet concentrates.

Parameter	all	COD	WGT	P-Value	Low vWF:Ag (≤425%)	High vWF:Ag (>425%)	P-Value	Low vWF:Ag/ COD	High vWF:Ag/ COD	Low vWF:Ag/ WGT	High vWF:Ag/ WGT	P-Value
Patients (n)	31	20	11	-	16	15	-	11	9	5	6	-
RBC / ECMO day (n)	0.4 (0.2-0.6)	0.4 (0.2-0.4)	0.5 (0.3-0.8)	n.s.	0.4 (0.2-0.5)	0.4 (0.2-0.7)	n.s.	0.4 (0.2-0.4)	0.4 (0.2-0.6)	0.5 (0.5-0.7)	0.5 (0.3-0.7)	n.s.
PC / ECMO day (n)	0.0 (0.0-0.1)	0.0 (0.0-0.0)	0.0 (0.0-0.2)	n.s.	0.0 (0.0-0.1)	0.0 (0.0-0.0)	n.s.	0.0 (0.0-0.0)	0.0 (0.0-0.3)	0.1 (0.0-0.2)	0.0 (0.0-0.0)	n.s.
FFP / ECMO day (n)	0.0 (0.0-0.01)	0.0 (0.0-0.1)	0.0 (0.0-0.0)	n.s.	0.0 (0.0-0.1)	0.0 (0.0-0.0)	n.s.	0.0 (0.0-0.1)	0.0 (0.0-0.0)	0.0 (0.0-0.0)	0.0 (0.0-0.0)	n.s.

n.s., not significant

RBC, red blood cells; FFP, fresh frozen plasma (1 FFP contains 230mL plasma); PC, platelet concentrate (1 PC contains 250mL and 2 – 4 x 10^{11} thrombocytes).
